# Undergraduate e-learning programmes in health professions: An integrative review of evaluation standards in low- and middle-income countries

**DOI:** 10.1371/journal.pone.0281586

**Published:** 2023-02-13

**Authors:** Moses M. Mutua, Champion N. Nyoni

**Affiliations:** Faculty of Health Sciences, School of Nursing, University of the Free State, Bloemfontein, South Africa; Ege University Faculty of Medicine, TURKEY

## Abstract

**Background:**

Before the Coronavirus COVID-19, universities offered blended learning as a mode of study. However, with the closure of all educational institutions, after the pandemic, most of these institutions were required to transition to e-learning to support continuous student learning. This transition was challenging to most institutions, as there were no standards to ensure the quality of e-learning. During this literature review, the researcher aimed to explore relevant literature and provide insight into the standards for undergraduate e-learning programmes in the health professions.

**Design:**

An integrative review of literature.

**Data sources:**

Online databases MEDLINE, CINAHL with full text, Academic search ultimate, APA PsycInfo, ERIC, Health Source: Nursing/academic edition, CAB abstracts, Africa-wide information, Sociology source ultimate, and Communication and Mass media complete were searched.

**Materials and methods:**

Studies pertaining to low- and middle-income countries (LMICs) on standards in evaluating undergraduate e-learning programmes in health professions, published between January 2010 to June 2022, were considered. A two-step process was followed involving three reviewers and guided by an inclusion criteria focused on the evaluation of undergraduate e-learning programmes in the health professions. The initial hit produced 610 articles altogether, and eight articles that met the inclusion criteria were included in the study. Data was then extracted and analysed, and key themes were identified.

**Results:**

Eight Key themes related to LMIC standards emerged from the eight selected articles: curriculum planning, proficiency of educators, learner proficiency and attitude, infrastructure for learning, support and evaluation.

**Conclusion:**

In this review, we synthesised standards that have been used for evaluating undergraduate e-learning programmes in health professions in LMICs. A gap in standards related to clinical teaching and learning in undergraduate e-learning programmes in the health professions was evident from all the included articles. The identification of the eight unique LMIC standards in this review could contribute to guiding towards contextually appropriate quality e-learning programmes in the health professions.

## Introduction

Employers are sceptical about hiring health professionals whose qualifications are obtained through e-learning [1]. Clinical incompetence and poor quality of assessments are often used to support arguments against the employment of such health professionals [2]. These narratives are palatable to the regulators of health professions, who often argue in line with protecting the public from poorly trained and incompetent health professionals [1–3]. However, technological advancements and the recent COVID-19 pandemic have effectuated the rapid uptake of e-learning and distance education into mainstream programmes in the health professions. Strategies need to be in place to evaluate and guarantee quality in these programmes. The focus of this review is on the theoretical components of training health professions inferences are made, however, on the need for integrating clinical practice on e-learning platforms, albeit being a challenge in LMICs.

An educational programme is evaluated against standards to determine its quality [1, 3, 4]. The literature describes various models and frameworks used for assessing the quality of e-learning programmes. The quality matters (QM) model is a prominent model for evaluating e-learning quality and has generated widespread interest through several studies that support its impact [4]. The QM model applies eight standards: the course overview, learning objectives, assessment and measurement, resources, materials, learning engagement, course technology, learner support, and accessibility in evaluating the quality of e-learning programmes. The context, inputs, processes and product (CIPP) model was initially developed by Stufflebeam [5]. Fishbain et al. [6] operationalised it in describing the process of evaluating online programmes, considering the structure, complexity, and cultural differences. Furthermore, essential elements in evaluating the quality of e-learning programmes such as technological infrastructure, institutional support, course design, support of instruction and learning, assessment, educators, administration, and curricular structure have been reported [4, 7, 8]. Fishbain et al. [6] make a futuristic recommendation related to evaluating e-learning programmes for the health professions by including patient outcomes. The above models and frameworks present a plethora of approaches used to evaluate e-learning programmes reflecting various areas of chiasm and divergence. However, the operationalisation of these models and frameworks within undergraduate e-learning programmes in health professions education is complex.

Undergraduate education programmes in the health professions integrate clinical and theoretical components [1]. The clinical detail adds an absorbing layer of complexity in evaluating and maintaining quality in an e-learning programme. The clinical environment where students learn is often distant from the online educator, who relies on the clinicians to support students to develop clinical competence [1, 9]. Kolb and Kolb [10] argue that classroom activities must align with the clinical experience to enhance learning and avoid cognitive dissonance, which interferes with learning and hinders competence development [11]. Thus, students enrolled in undergraduate e-learning in the health professions programmes may experience cognitive dissonance in the clinical setting due to differing resources, approaches to healthcare, disease profile, student support and online educators who may apply different standards. The literature confirms the influence of cognitive dissonance on the perceptions of the quality of educational programmes [12, 13]. Therefore, more specific standards and approaches in determining and ensuring quality in e-learning nursing education may be needed in health professions programmes to learn and demonstrate skills and competencies [1]. However, at the time of Delva et al. ‘s [1] study, there were no reported standards specific to evaluating the quality of undergraduate nursing programmes and no reported entity in the United States responsible for the oversight and continuous evaluation of the quality of online nursing programmes. The unavailability of explicit strategies for evaluating e-learning undergraduate programmes in the United States may be similar in other countries, including Low- and middle-income countries (LMICs). The uniqueness of LMICs is that their challenges are dissimilar from developed countries. Hence context-specific standards will enhance the quality of e-learning.

E-learning provides greater flexibility concerning time, space, language, content, and administrative power than traditional face-to-face learning [14]. Technological advances in various areas, including education in the health professions, and the integration of e-learning and online distance education, is inevitable. However, there is a need to understand the determinants of quality standards in e-learning programmes in the health professions underpinned by the complexity brought on by educators, and students, variations in resources, regulations, and clinical outcomes. This article presents an integrative review that synthesised evidence related to standards used to determine quality in undergraduate e-learning programmes in the health professions from LMICs. We argue that low-resource settings contribute to the discourse related to evaluating quality in undergraduate e-learning programmes in the health professions, which could guide the development of contextually appropriate interventions. Such interventions need to enhance quality and should ensure the regulators and the public of the competence of health professionals trained through e-learning programmes. The purpose of the current review was to synthesise available literature related to standards for evaluating undergraduate e-learning programmes in the health professions in LMICs. The outcomes of this review will be standards that will be used in LMICs to evaluate the quality of e-learning. These standards will then be used to evaluate the quality of e-learning in undergraduate e-learning nursing programmes in LMICs.

## Materials and methods

This section seeks to present the approach used in the integrative review for evaluating undergraduate e-learning programmes in the health professions in LMICs. The purpose of an integrative review is to summarize what is known about a topic and communicate the synthesis of literature to a targeted community [16, 17]. An Integrative review synthesizes research and draws conclusions from diverse sources on a topic and provides a more holistic understanding of a specific phenomenon with a focus on current state of evidence of a particular phenomenon [17]. A systematic review differs from an integrative review because it has a single narrow focused clinical question formulated in a PICO format and may include Meta analysis and requires protocol registration [16].

### Design

An integrative review approach included theoretical and empirical literature on varied methodologies and designs [15–17]. In the sections below, we report the five main steps of the integrative review:

### Problem identification

The following research question guided this integrative review:

‘Which standards are used in evaluating undergraduate e-learning programmes in the health professions in LMICs?

### Literature search

The research question was operationalised by identifying keywords and their synonyms linked through Boolean operators and modifiers to generate a search string. The keywords were ‘evaluating’, ‘e-learning’, and ‘health professions’. After being piloted, the search string was refined through a ‘quick and dirty’ search by an information specialist. The final search string in this review was:

(assess* evaluat* measur* licen* accredit*) n3 (“electronic teaching” “online education” “internet-based learning” “blended education” e-learning “Online learning” “Distance learning” “Blended learning” “Computer-assisted instruction” “Computer-assisted learning”) (Policy policies Benchmark* Qualit* criter* Principle* Descriptor* “quality standard*”) (“health science*” Nurs* medic* healthcare)

The search string was used to search for the literature through a university library. The EBSCOhost interface was used to access databases for this review. The following databases generated 610 hits: MEDLINE, CINAHL with full text, academic search ultimate, APA PsycInfo, ERIC, Health source: Nursing/academic edition, CAB abstracts, Africa-wide information, Sociology Source Ultimate, and Communication and Mass Media Complete. The hits generated are presented as titles and abstracts.

### Data extraction

An author-generated custom-made Google form was used to extract data from the included articles. The form was piloted on two articles by both authors and refined to their satisfaction. The characteristics of the articles were author name, the title of the study, and country, but also the purpose of the study, population group, the study design and results or outcomes which were also identified from the included articles. The evaluation models, data collection and data analysis methods, as well as recommendations and limitations, were identified (Table 1). The first author extracted data from the included articles, and the second author checked the work and resolved discrepancies through discussions.

**Table 1:** Data extraction table.

Timestamp	Number/Unique identifier	Author	Title	Year	Country	Population	Design	Result/Outcome	Evaluation Model used	Data collection method	Recommendation	Limitations	Purpose of the study	Column1	Data analysis Method	Population 2	AGE
7/20/2021 8:30:40	1	Ali Akbar Khosraavi Babadi, Fatemeh Yousefi Ehraz	The Feasibility of Using Blended Learning in the Curriculum of Speech Therapy at Tehran University of Medical Sciences	2018	Tehran	The subjects consisted of 48 undergraduate students, 25 undergraduate students, 22 PhD. students, and six faculty members selected by random sampling method using Morgan table. Faculty members were also counted pp 163	The current study employed the survey method, which is among the quantitative methods. pp 165	it can be concluded that both the faculty members and the students selected the options, few and very few for the current situation. In other words most of them stated that in the field of speech therapy the Blended Learning is rarely used at present. pg 165		The measuring tool was the questionnaire prepared with six indicators. Need Assessment, educational goals, learning, content and the curriculum course plan, equipment and infrastructures, and the evaluation pp165	According to the existence of practical courses in this field and the necessity to implement practical techniques on the patient, environments similar to real situations should be created to acquaint learners with real situa-tions that requires strengthening the infrastructures of equipment, providing physical environments, training experienced manpower, and supplying electronic content including the content quality, supporting the learners, inclining and motivating the learners; the learners characteristics, the flexibility of time and place in the education process, access to updated resources, interests of the learners, and the processes related to it should also be considered pp 167.	nad					
7/20/2021 13:20:21	2	Mohamad Jebraeily, Habibollah Pirnejad, Aram Feizi and Zahra Niazkhani	Evaluation of blended medical education from lecturers’ and students’ viewpoint: a qualitative study in a developing country	2020	Iran	The participants in our interviews consisted of 11 faculty members and 13 masters’ and professional doctorate students at the UUMS that had used the blended education in more than four university courses during at least two educational semesters in 2017–2018 pp 3	This study is a qualitative research conducted at the UUMS in 2018 using semi-structured interviews and the documents associated with the development and implementation of the blended learning available at the university and the deputy of education at the Iranian ministry of health and medical education, pp 3	Strengths • Focus on students’ learning needs • Matching with students’ interests in digital tools • Flexibility • Variety of teaching and evaluation methods • Improving productive lecturer-student interactions • Improving self-learning and problem-solving skills. Weaknesses • Lack of training courses • Deficiency in IT and network infrastructures and human resources • Lack of institutionalization of virtual education culture • Lack of an independent LMS • Complex and time-consuming task of virtual education • Lack of sufficient suitable feedback • Lack of information and reminder • Lack of required infrastructure and skillful human resources. Opportunities • Health education transformation and innovation plan of the ministry of health and medical education • The university’s and ministry’s senior executives support • Foundation of virtual university of medical sciences • User-friendly and easy-to-use LMS to be acquainted with • Access to the updated and standard electronic contents • Development of regional and international cooperation. Threats • Dependence of virtual education development on the national education transformation plan • Resistance of the lecturers to use the virtual education • Lack of proper evaluation of virtual education • Non-compliance with the principles of copyright and intellectual property • Insufficiency of the privileges considered at the university for people who use the virtual education • Lack of an independent virtual education center at the university.	To evaluate such issues while implementing an intervention, the strengths, weaknesses, opportunities, and threats (SWOT) analysis is a useful method. This method aims to recognize strengths and weaknesses in inner dimensions of an organization and opportunities and threats in outer dimensions, pp 2.	This study is a qualitative research conducted at the UUMS in 2018 using semi-structured interviews and the documents associated with the development and implementation of the blended learning available at the university and the deputy of education at the Iranian ministry of health and medical education, pp3.	in this study provide insights for policymakers Jebraeily et al. BMC Medical Education (2020) 20:482 Page 9 of 11 and university executives regarding the management strategies for a smooth transition towards blended education and its adoption in order to reap the full benefits, Therefore, if the SWOT items are recognized and considered mindfully in each context, they can help to adopt the right implementation and management strategies to achieve sustainable benefits, pp 10	NAD					
7/21/2021 10:18:33	3	Atekeh Mousavia, Aeen Mohammadia, Rita Mojtahedzadeha, Mandana Shirazi and Hamed Rashidi	E-learning educational atmosphere measure (EEAM): a new instrument for assessing e-students’ perception of educational environment	2020	Iran	We thoroughly presented factors creating educational atmosphere in an e- learning setting to a committee comprising five e-learning and education experts who devised the draft of an instrument for its assessment. The draft was reviewed using the comments of other 10 experts in e-learning and education to assess its face validity. the instrument was presented to 185 students studying virtual programmes by e-mail or in-person pp 4	qualitative design	The expert committee devised 43 items as instruments’ questions that they believed would cover assessing educational atmosphere in an e-learning environment. All the questions were designed in the 5-point Likert scale, including totally agree, agree, neutral, disagree and absolutely disagree. The final instrument, named as ‘e-learning Educational Atmosphere Measure’ (EEAM), included 40 questions that covered six factors consisting of programme effectiveness, teaching quality, ethics and professionalism, learner support, safety and convenience, and awareness of the rules pp 5.		The final instrument, named as ‘e-learning Educational Atmosphere Measure’ (EEAM), included 40 questions that covered six factors consisting of programme effectiveness, teaching quality, ethics and professionalism, learner support, safety and convenience, and awareness of the rules pp5.	This tool is suitable for current e-learning courses which are interactive ones and apply a wide range of synchronous and asynchronous strategies for enhancing teaching–learning processes. EEAM has the advantage of covering relevant aspects and factors explored in previous studies together with new ones, which makes it a comprehensive tool. Using this instrument, educators would explore methods to improve their e-classes based on their students’ perception, leading to enhancing learning outcomes pp 6.	nad					
7/21/2021 11:05:08	4	Pracheth Raghuveer, Abhay Subhashrao Nirgude	Utility Of E-learning in Community Medicine: A Mixed Methods Assessment Among Indian Medical Students	2016	India	total of 323 undergraduate medical students participated in the study, of which 164 (50.77%) were males and 159 (49.23%) were females pp 90	We adopted a cross-sectional study design using a mixed methods approach involving both quantitative and qualitative elements to fulfill the study objectives pp 89	Almost all the study participants owned a personal computer/laptop (99.07%) and 98.76% had access to the Internet 63.77% reported the use of internet several times a day. A high proportion (66.87%) of the study participants spent < 1hr online carrying out academic activities and 37.77% spent < 1 hr online on leisure activities. A high percentage (82.35%) stated that they were confident overall in using a computer. wide majority (81.74%) reported that they are self-proficient in using e-learning. When the usefulness of different e-learning activities were assessed, 67.80% found power-point presentations and descriptive texts pertaining to the lecture topics taught, useful. Most of the study participants agreed that e-learning is useful (62.29%). More than half (52.63%) reported that e-learning improves the teaching standard.	Qualitative assessment was carried out by Force Field Analysis (FFA), a technique developed by Kurt Lewin, employed to identify and analyze the forces affecting a problem situation, so as to bring about a change.8 FFA is an important tool which facilitates the diagnosis of situations and controlling changes within organizations and communities pp89	This study was carried out in 2 phases, phase 1, a questionnaire-based quantitative assessment and phase 2, a qualitative assessment	Formal orientation programmes for students are the need of the hour. Provision of structured computer and information technology training for medical students would equip them with the skills they need to utilize e-learning in a more desirable manner pp 92. To enhance the judicious use of e-learning, training for faculty is a must. Moreover, e-learning may be used as a platform to sensitize medical students about the basics of clinical examination and certain important medical procedures by uploading relevant videos, images and illustrations. A judicious use of e-learning may certainly act as a supplement to the conventional classroom teaching pp92	The cross-sectional nature of our study limits the assessment of the effectiveness of e-learning among the undergraduate medical students pp 92					
7/31/2021 7:41:22	5	Azizeh K. Sowana,*, Louise S. Jenkins	Designing, delivering and evaluating a distance learning nursing course responsive to students needs	2013	Jordan	Participants included all undergraduate nursing students who were enrolled in a distance and a hybrid section of a communication skills course offered at a School of Nursing in Jordan. The final sample included 25 students in the distance section and 35 in the hybrid section (N = 60)pp 553.	The study followed a mixed methodology design where the intervention group included students who were enrolled in the distance class (n = 32) and the control group composed of students in the hybrid class (n = 28 students). The “qualitative” part of the study design included using open-ended questions in the satisfaction questionnaire pp 555	At the first two weeks of the course, seven of the distance students transferred to the hybrid class because of the perceived difficulty to manage the course using this new method of learning. Five of these students were bridging students who had a nursing diploma and were pursuing their Bachelor’s degree in nursing. This resulted in a total of 25 students in the distance class and 35 in the hybrid one. The majority of students in the two classes reported that they had not used Blackboard or discussion board questions in previous courses, and had a computer at home but with no internet connection. The percentage of students who used e-mail for educational purposes in the distance class had increased from 12% before this course to 60% after the course. Also, in the distance class, 72% of the students (18) reported that they listened to video sessions using the course CD rather than Blackboard via their home computers pp 558	nad	For the purpose of this study, a 27-item questionnaire was designed to assess satisfaction of the distance student with the course. The questionnaire was developed based on extensive review of different studies in online education and the Framework and Benchmark for Best Practice in Web-based Nursing Courses [17]. The questionnaire was targeted to gain information from a student perspective about the course design and delivery processes, their online experience, quality of assignments, active and collaborative learning experiences, and instructor support. Student responses were elicited using a 5-point Likert scale, ranging from strongly agree (5) to strongly disagree (1). In addition, two open-ended questions about the advantages and disadvantages of distance education were asked to capture more individualized perceptions of the distance experience pp 558	The use of effective instructional strategies resulted in delivering successful distance learning, even for students with limited resources. • Assessing student characteristics in distance learning is imperative to tailor course design and delivery processes in order to optimize the use of technology to fit students’ individualized learning needs. • Special attention should be directed toward helping students manage their time in distance learning environments and work effectively in group assignments pp 563.	Potential limitations of this study include the relatively small convenience sample size of students, the lack of random assignment to groups, and the high proportion of male to female students, which limit generalizability. In addition, since the first author taught both sections, this might introduce some bias. The high inter-rater agreement between the first author and another faculty member on scoring the course assignments was used to address this bias. pp 562	the current study describes the design and delivery processes, and experience of undergraduate nursing students in the first distance course offered at a school of nursing in Jordan pp 554		SPSS version 16.0 (SPSS Inc., Chicago, IL) was used for statistical analyses. Statistical methods were conducted using a significance level of 0.05 and included descriptive analyses of means and standard deviations for the satisfaction questionnaire; chi square, Fisher’s Exact test and t-test to measure difference in student characteristics between the distance and hybrid classes; t-test for difference in achievement between the distance and hybrid classes; and appropriate tests (t-test and Pearson’s r) to examine the difference in means and relationships between satisfaction and other variables based on the level of measurement pp 558.		
8/1/2021 22:34:20	6	Ismail M. Saiboon, Fareena Zahari, Hisham M. Isa, Dazlin M. Sabardin and Colin E. Robertson	E-Learning in Teaching Emergency Disaster Response Among Undergraduate Medical Students in Malaysia	2021	Malaysia	A prospective cross-sectional study among pre-clinical yearmedical students was carried out to determine their knowledge on DRM and perception regarding the ELITE-DR initiative using a validated online questionnaire, A total of 168 students participated in the study pp 1	This was a prospective, cross-sectional, interventional study involving pre-clinical year students at Faculty of Medicine Universiti Kebangsaan Malaysia (UKM), looking at pre- and post-intervention outcomes pp2	From a total of 274 undergraduate medical students in the preclinical year eligible for this study, 261 participants consented and were enrolled into the study. Thirteen were excluded from the study, 12 were because they had attended a disaster response medicine course prior this and one declined to participate. A total of 93 participants did not complete the study; a final total of 168 participants completed the study, Of the 168 participants, the majority were females from the first year (N = 135; 80.4%). Most of the participants (86.3%) watched the SLVs only once.	nad	The study collected quantitative data which included the participants’ knowledge and perceptions. Respondents were invited to answer a self-administered questionnaire. Twenty questions assessed knowledge, while 26 questions assessed perceptions. Questions assessing knowledge were divided into principles and medical management of DRM. The knowledgebased questions were multiple-choice questions with a single best answer, and each question carried one (1) mark. The 26 items on the perceptions comprised the self-gain, presentability, and e-learning in the medical curriculum. These questions were developed by a group of three expert panels from among the local emergency physicians who specialized in DRM	study revealed that ELITE-DR, a novel e-learning platform is beneficial in teaching-learning of emergency DRM among UG medical students. Recall of knowledge comprehension and simple analysis-application for basic decision-making was particularly well-served through ELITE-DR, whereas complex decision-making knowledge aspects such as treatment and transport decisions were likely to require a different approach, perhaps one that incorporates feedback	The number of questions in the questionnaires is relatively low and might not be adequate in assessing some topics. Nevertheless, our study gives a general idea of the capability of video teaching in acquiring factual knowledge and decision making in DRM. This study did not explore or evaluate the psychomotor skills that students would usually perform during F2F simulation exercises of DRM, including Airway, Breathing, Circulation, and Immobilization procedures. These include standard first aid procedures (bleeding and wound management, splinting, and bandaging), invasive procedural skills (airway and ventilation management, intravenous cannulation), carrying and lifting of victims. We did not evaluate the relative time spent by the participants in watching the three videos. This might help reveal whether the duration of the video watching relates to the effectiveness of learning and knowledge retention. The study also did not compare synchronous (real-time interaction using any online application like Zoom, Microsoft Teams, etc.) with asynchronous teaching (not real-time interaction) of DRM. Our study did not compare with the other technique of disaster response medicine teaching because most of the time this subject were taught using a classroom medium either as a face-to-face immersive simulation, table-top exercise or hybrid simulation of approach combining e-learning with classroom immersive simulation. this study did not incorporate debriefing even though debriefing is a very powerful learning tool because debriefing was not suitable for an asynchronous methods of teaching. Furthermore, debriefing after watching the video would disrupt the findings of the study.	We evaluate the effectiveness of e-learning in teaching emergency disaster response (ELITE-DR), a novel initiative, in educatingmedical students of the cognitive aspect of DRM.		Statistical analyses were performed using Minitab Statistical Software (version 19, Pennsylvania State University, PA, USA). Demographic characteristics of the participants were obtained by descriptive analysis. Data were summarized using mean and standard deviation for continuous variables, frequency, and percentages for categorical variables. The 99% confidence interval was calculated for the mean scores. A paired t-test was used to assess the mean differences between the two groups (knowledge between pre- and post-test), and one-way ANOVA was used to compare the PS achieve among the four groups of PS. All differences were considered statistically significant if p < 0.01 pp 5		
	7	Nourhan F. Wasfy1, Enjy Abouzeid1, Asmaa Abdel Nasser1,2, Samar A. Ahmed3, Ilham Youssry4, Nagwa N. Hegazy5*, Mohamed Hany K. Shehata6,7, Doaa Kamal1 and Hani Atwa1,7	A guide for evaluation of online learning in medical education: a qualitative reflective	2021	Egypt	To develop a set of descriptors for best practice in online learning in medical education utilizing existing expertise and needs.	faculty members, medical educators	qualitative design	development of a set of standards, indicators, and development of a checklist for each standard area. The standard areas identified were organizational capacity, educational effectiveness, and human resources each of which listed a number of standards								

## Data analysis

This integrative review was underpinned by the contemporary framework for integrative reviews, which was applied throughout the process of analysing data [17]. Whittemore and Knafl [17] suggest a multi-step process for analysing data for an integrative review. Frequencies related to study characteristics were tallied, while an inductive thematic approach was used to identify standards for evaluating e-learning as presented by the included articles (Table 2).

**Table 2:** Data analysis table.

Author	Standard used in evaluating e-learning (theme)
**Babadi and Ehraz (2018)**	Needs assessment, educational goals, learning, content, curriculum course plan, equipment, infrastructures, evaluation, motivation of learners, interest of learners, stimulation for learners
**Jebraeily et al. (2020)**	Focusing on learning needs, flexibility of the virtual component, variety of teaching and evaluation methods, improved self-learning and problem-solving skills, improved lecturer–student interactions, matching with student interests in digital tools, IT and network infrastructure, human resources, institutionalisation of virtual education, independent learning management system, sufficient suitable feedback, develop regional and international cooperation, health education and transformation plan, administrative support, compliance with copyright and intellectual property
**Mousavi et al. (2020)**	Programme effectiveness, teaching skills, professionalism and professional ethics, learner support, safety and convenience, learning academic meta-skills, educator training
**Raghuveer and Nirgude (2016)**	Pattern on computer and internet usage, self-perceived computer skills, proficiency of e-learning use, attitudes on e-learning, usefulness of various e-learning resources, such as teaching and learning resources, easy access to learning material, accessibility outside campus, variety in content material to include videos, clinical scenarios, demonstration, and illustrations
**Sowan and Jenkins (2013)**	Course design, delivery process, student satisfaction, quality of instruction, active and collaborative learning experiences, instructor support
**Saiboon et al. (2021)**	Higher flexibility, personalised learning, usefulness, ease of use, perception of scores in e-learning affects knowledge scores
**Wasfy et al. (2021)**	Organizational capacity, resources, organizational bylaws, effective learning and assessment, course design, course delivery, student assessment, evaluation, human resources (faculty, students, and administration), digital tools, academic counselling
**Saleh et al. (2022)**	Online learning modality, the significance of Diploma, course content, instructors, transfer of learning into performance, personal development

The identified standards were further synthesised through pattern coding to reflect broader themes and domains related to evaluating e-learning in undergraduate programmes in the health professions.

## Results

### Selecting the articles

**Fig 1:**
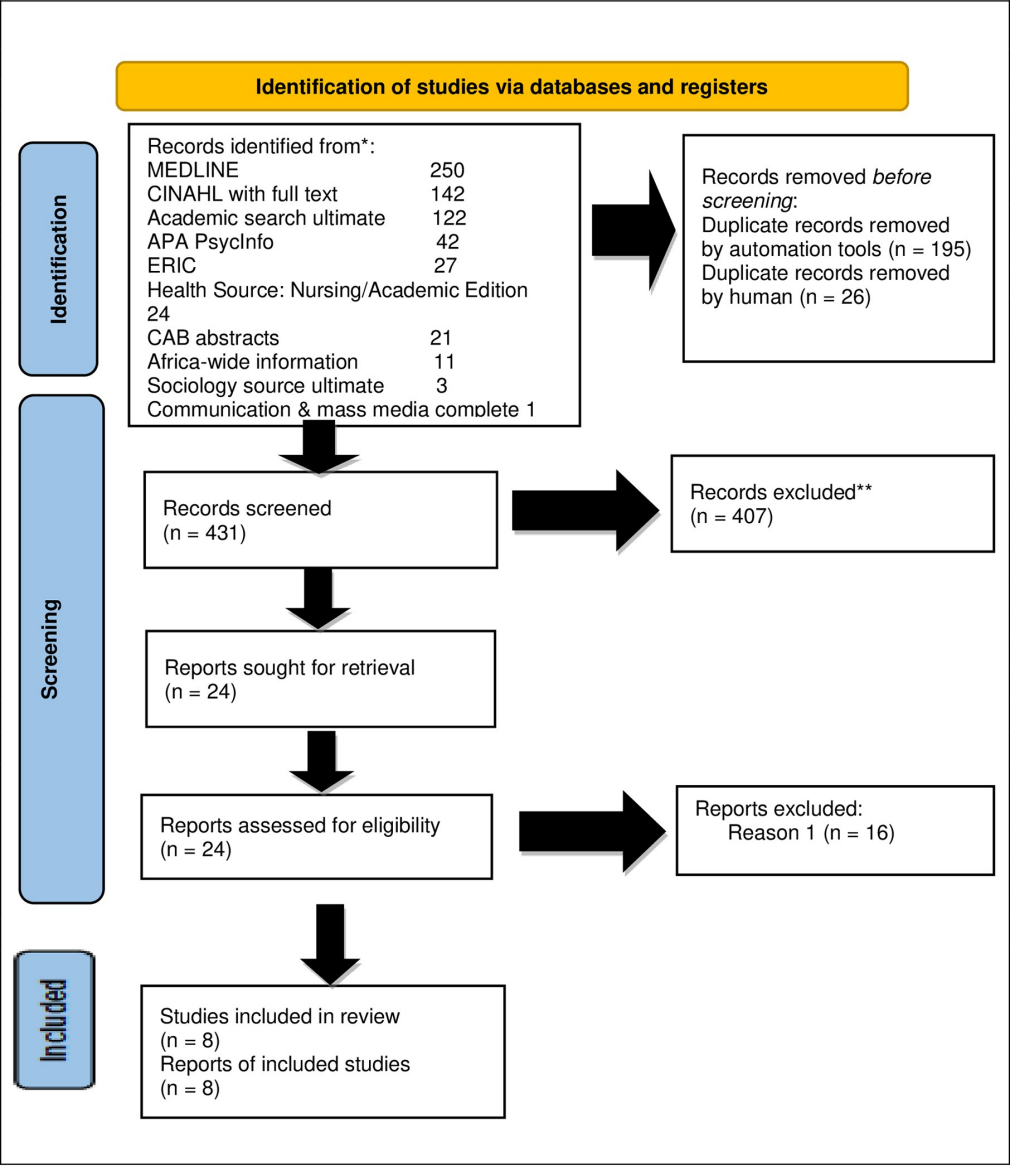
PRISMA (modified) flow diagram.

A two-step process involving three reviewers and guided by an inclusion criterion was applied in selecting articles to be included in this review. The two authors and an expert in the education of health professions from a low-income country screened and selected the articles. The study had the following, inclusion criteria focused on the evaluation of undergraduate e-learning programmes in the health professions, should have been published between January 2010 and December 2020 and had to be from LMICs. The literature was excluded from the review based on the following criteria. Literature not related to health sciences, literature on post-graduate programmes, Literature not accessible from the university of the Free State library and Literature dated before 2010. Integrative reviews summarise the latest research, and ten years may be sufficient to provide information that would be useful for future use. The purpose of the criteria was to identify the most appropriate articles to enable us to develop valid and reliable answers to research questions. The primary search strategy identified 610 studies, from which 205 duplicates were excluded through automatic and physical deduplication. Of the remaining 295 records, 269 were eliminated for various reasons, including inappropriate context, different subjects, including inappropriate context, different subjects, articles addressing post-graduate education, articles not from LMICs, article abstracts that the librarian could not get full articles and articles not written in English. Full texts articles were sought from 24 remaining records, of which 16 were eliminated as they did not meet the inclusion criteria. A total of eight articles met the criteria and are included in this review (Fig 1).

### Study characteristics

Based on the studies’ characteristics, three articles from Iran were selected. The other articles were from India, Jordan, and Malaysia. There was a paucity of articles from Africa and South America, where many countries are classified in the low- and middle-income brackets. Most of the studies (n = 3) were published in 2020 and 2021. One article reported on an undergraduate nursing programme [18], while the other three journals reported on an undergraduate medical programme [19]. Only two studies mentioned using models to underpin their evaluation processes, namely, the SWOT analysis [20], the Kirk Patrick model [33] and the force field analysis [21]. Most of the studies applied mixed methods research [18, 21, 22], while others used quantitative methods [18, 23] and qualitative methods [20]. Seven domains, each with a specific theme, were generated inductively after engaging with the individual themes from each included article [32]. Seven domains, each with a specific theme were generated inductively after engaging with the individual themes from each included article. The seven domains are presented in Table 3.

**Table 3:** Domains in the evaluation of e-learning.

*Domains*	Themes
***Curriculum planning***	Curriculum course plan and course design (Babadi and Ehraz, 2018)
	Quality of assignments (Sowan and Jenkins, 2013)
	Course content (Babadi and Ehraz, 2018)
	Improve self-learning and problem-solving skills (Jebraeily et al., 2020)
	Education goals and outcomes (Babadi and Ehraz, 2018)
	Learning academic meta skills (Mousavi et al., 2020)
	Course specifications linked to the methodology of the online teaching and the method of assessment (Wasfy et al., 2021)
	Written policies & procedures of all online courses (Wasfy et al., 2021)
	Course content comprehensive and inclusive (Saleh et al., 2022)
***Proficiency of educator***	Self-perceived computer skills (Raghuveer and Nirgude, 2016)
	Needs assessment (Babadi and Ehraz, 2018)
	Professionalism and professional ethics (Mousavi et al., 2020)
	Matching with student interests in digital tools (Jebraeily et al., 2020)
	Perception (Saiboon et al., 2021)
	Attitudes on e-learning (Raghuveer and Nirgude, 2016)
	Teaching skills (Mousavi et al., 2020)
	Training educators (Mousavi et al., 2020)
	Delivery process (Saiboon et al., 2021)
	Clarity on faculty roles and responsibilities (Wasfy et al., 2021)
	Programme for continuous faculty training on skills for online teaching (Wasfy et al., 2021)
	Assigned faculty numbers are reasonable, sufficient, and aligned with the student number and educational activities (Wasfy et al., 2021)
	Instructors provide constructive feedback in a timely manner (Saleh et al., 2022)
***Learner proficiency and attitude***	Lecturer-student interactions (Jebraeily et al., 2020)
	Active and collaborative learning experiences (Sowan, and Jenkins, 2013)
	Variety of teaching and evaluation methods (Jebraeily et al., 2020)
	Personalised learning (Saiboon et al., 2021)
	Student satisfaction (Sowan and Jenkins, 2013)
	Focus on learning needs (Jebraeily et al., 2020)
	Interest of the learners (Babadi and Ehraz, 2018)
	Established programme for student training (Wasfy et al., 2021)
	Plan for academic counselling that is clear, manageable, and executed (Wasfy et al., 2021)
	Learner confidence because of proper guidance in teaching (Saleh et al., 2022)
***Infrastructure for learning***	Equipment (Babadi and Ehraz, 2018)
	Accessibility outside campus (Raghuveer and Nirgude, 2016)
	Easy access to learning materials (Raghuveer and Nirgude, 2016)
	Flexibility of the virtual component (Jebraeily et al., 2020)
	Ease of use of materials (Saiboon et al., 2021)
	IT and network infrastructure and human resources (Jebraeily et al., 2020)
	Independent learning management system (Jebraeily et al., 2020)
	Infrastructure and skilful human resource (Jebraeily et al., 2020)
	Compliance principle and intellectual property (Jebraeily et al., 2020)
	Ease of use (Sowan and Jenkins, 2013)
	Variety of content material to include: videos, clinical scenarios, demonstrations, illustrations. (Raghuveer and Nirgude, 2016)
	Usefulness of various e-learning resources such as teaching and learning resources (Raghuveer and Nirgude, 2016)
	Financial resources allocated to online learning (Wasfy et al., 2021)
***Support***	Learner support (Mousavi et al., 2020)
	Instructor support (Sowan and Jenkins, 2013)
	Institutional plan and policy (Raghuveer and Nirgude, 2016)
	Administrative support (Jebraeily et al., 2020)
	Trained technical support team (Wasfy et al., 2021)
***Evaluation***	Pattern on computer and internet usage (Raghuveer and Nirgude, 2016)
	Student online experience (Mousavi et al., 2020)
	Effectiveness of programme (Mousavi et al., 2020)
	Safety and convenience (Mousavi et al., 2020)
	Monitoring and evaluation for the online learning materials/ process by internal reviewers for continuous improvement (Wasfy et al., 2021)
	Evaluation data drives decisions for continuous improvement (Wasfy et al., 2021)

As expected, a relatively low number of articles were included in this review. This finding could be attributed to the low uptake of e-learning programmes in low-resource settings versus the resource-intensive nature of e-learning programmes [24–26]. Brooks et al. [27] further explain that the scepticism around the quality of health profession graduates from e-learning or distance learning programmes hampers national investments in e-learning programmes, with educational institutions preferring face-to-face programmes. In the same vein, research outputs from low-resource settings are often low; hence there are limited publications in general [2].

## Discussions

The purpose of this review was to synthesise available evidence related to standards for evaluating undergraduate e-learning programmes in the health professions in LMICs. The results of the integrated review of standards presented characteristics of the eight studies. They then focused on the seven domains related to the evaluation of undergraduate e-learning programmes in the health professions in LMICs.

The seven evaluation-related domains were curriculum planning, proficiency of the educator, learner proficiency and attitude, infrastructure for learning, support, and evaluation (data in S1 Text). There are similarities between the domains from this review and some of the popular standards used in high-income countries [28]. The similarity in these standards may be due to models and theories related to evaluation that are often transferred from high-income countries to low-income counties under the guise of confirmed validity [20, 22]. In addition, there are reported similarities among undergraduate e-learning programmes in the health professions between high- and low-income countries, supporting the findings from this review on the similarity of evaluation standards.

The domain related to curriculum planning encompasses various aspects of the structure of e-learning programmes. Babadi and Ehraz [23] reflect on the curriculum course plan and course design, educational outcomes and goals, and course content, while Sowan and Jenkins [15] describe assignments as part of curriculum planning. Wasfy et al. [32] describe course specifications linked to the methodology of online teaching and the method of assessment with well-formulated policies and procedures for online courses as desired requirements for e-learning programmes. This domain for evaluating undergraduate e-learning programmes in the health professions in LMICs is aligned with the course overview and standards of learning objectives as defined by the QM model [1]. The domain further aligns with the curricular structure of Capacho et al. [7] and the course design [8]. Prideaux [29] notes three levels of a curriculum: planned, prescribed, or implemented curriculum, which the former is often operationalised in educational programmes through specific outcomes, goals, designs and even assessments. Therefore, it seems common that the structure of the programme–specifically the curriculum plan for an e-learning programme must be included as part of the standards for evaluating undergraduate e-learning programmes in the health professions.

The uptake of e-learning in undergraduate education in the health professions in LMICs has been hampered by the resource-intensive nature of such programmes [25, 30]. The second domain from this review relates to the infrastructure for learning. Several authors described the necessary infrastructure for learning as part of the evaluation standards for their e-learning programmes [20, 22, 23, 31]. This infrastructure included equipment, information technology (IT) and network infrastructure, an independent learning management system and skilful human resources. In addition, other authors focused on the ease of accessing and using a specific infrastructure for learning [20, 22, 23]. Raghuveer and Nirgude [22] further mention the need for a variety of content material to be included and for compliance with principles and intellectual property. Wafsy et al. [32] describe the need for financial resources allocated for online learning. Infrastructure for e-learning is aligned with resources, materials and course technology as described by the QM model, with technology as described by Capacho et al. [7] and with technological infrastructure Kumar et al. [4]. As e-learning is based on the integration of technology in an educational programme, the standards for evaluating e-learning should have a specific focus on technological resources and use within a programme. Insufficient resources for learning, typical in resource-limited settings, compromise the quality of e-learning programmes.

The proficiency of educators is reported as a domain in this study and focuses on the educators facilitating e-learning. Raghuveer and Nirgude [22] reported that educators must be proficient and competent in facilitating e-learning. Authors of all included studies evaluated the online teaching and delivery skills of educators [18–23]. Moreover, the requirement of educators related to e-learning, including their perception and attitude, was included as part of needs assessments. Additionally, an evaluation of professionalism and ethics during the delivery of content was reported as well as the ability of educators to match students’ interests with digital tools. The role of educators in implementing e-learning programmes successfully cannot be overemphasised, as educators have the potential to influence the programme negatively. As part of educator development, continuous training on skills for online teaching is essential [32]. Feedback provided by educators should be constructive and timely to foster efficient implementation by learners [33]. The proficiency of the educator domain identified in this study is similar to that reported by Capacho et al. [7], while the QM model and Kumar et al. [4] are silent regarding the educator’s proficiency. In LMICs, educators are often not well versed in advancements in educational technology and may be pressured to copy and paste face-to-face teaching modalities into the online or e-learning space. Strategies need to be designed and implemented that focus on determining educator needs, developing the educator, and continuously monitoring and evaluating educators on their effectiveness in the online space.

The learners enrolled in an e-learning programme must be proficient in learning through digital means and possess the appropriate attitudes [23]. In this review, the domain of learner proficiency and attitude reflected standards used to evaluate learners in the e-learning space. The sub-themes were active and collaborative learning experiences, personalised learning, focus on learning needs, and the interest and motivation of learners to learn through electronic means. Jebraeily et al. [20] further focused on the process of improved self-learning and problem-solving skills. The learner proficiency domain was not reported in the other popular models for evaluating e-learning [4, 7]. Learners are expected to have some degree of proficiency specifically related to the technology used in their learning. Babadi and Ehraz [23], as well as Sowan and Jenkins [18], state that orienting learners to the learning technologies and subsequent support or maintenance of proficiency is an essential element in student learning. Learner proficiency should be an indispensable standard where institutions must focus on the competence of learners regarding learning technologies. Furthermore, Raghuveer and Nirgude [22] mention the role and value of attitudes towards e-learning, as learners should have an interest in the learning approaches. Moreover, a programme for learner training and a plan for academic counselling that is clear, manageable, and executed is required [32]. Just as in the case of educators, the literature by Babadi and Ehraz [23] specifies that the uptake, use and usefulness of such technology are poor when the learners’ interest in the technology is limited, thus compromising the quality of e-learning. Therefore, standards for e-learning should evaluate learner interest in addition to learner proficiency.

Support is understood as specific strategies, techniques and approaches that assist the attainment of a specific outcome. In this review, the included articles reflected the application of support as part of the standards used in evaluating the quality of e-learning programmes. Learners and their educators are expected to be supported during the teaching and learning activities [18, 20, 21]. The support should be from trained technical teams [32]. The support is focused on learners, specifically relating to technical support [21] and instructor support [18]. Jebraeily et al. [20] further mention the role of administrative support and the need for support gleaned from institutional plans and policies that direct the nature and type of support for learners and educators. These findings are aligned with other models for describing quality in e-learning programmes. The QM model refers to learner support and accessibility as a standard for evaluating the quality of e-learning support. Capacho et al. [7] relate to support in general, while Kumar et al. [4] relate to support of instruction and learning and institutional support. All e-learning programmes should support learners, and in the evaluation of quality, the nature and type of support need to be made explicit and should be accessible for learners and educators. The support should facilitate quality e-learning within the expected contextual remits.

The last domain, which focuses on the evaluation, provides institutions, programmes, and educators with information on the engagement with learners and the value of their e-learning programmes. In evaluating the quality of e-learning programmes, studies included in this review reflected evaluation elements using various parameters. Raghuveer and Nirgude [22] relate to the pattern of computer and internet usage, while Mousavi et al. [21] comment on issues related to programme [effectiveness, safety, convenience, and student satisfaction [18]]. The integration of internal reviewers, to monitor online learning materials and processes, and the data generated from the reviews drives decisions for continuous improvement [32]. Only Kumar et al. [4] applied standards aligned with evaluation, namely effectiveness of learning and satisfaction of learners and educators. The standards related to evaluation emphasise an interplay of process and outcome monitoring [21, 22]. However, there are gaps related to the longitudinal outcome of undergraduate e-learning programmes in the health professions.

Studies included in this review reported a wide array of standards and indicators of quality within undergraduate e-learning programmes in the health professions [18–21, 23]. However, none of the included articles described evaluation standards related to teaching and learning of clinical skills within undergraduate e-learning programmes in the health professions. The lack of standards for evaluating teaching and learning clinical skills is a significant gap within the literature from LMICs [25]. The generic standards used in undergraduate e-learning programmes in the health professions often miss the intricacies of clinical education [1]. There is a need for the development and integration of standards for evaluating undergraduate e-learning programmes in the health professions that include the evaluation of clinical education. These standards would support education institutions in determining the quality of their programmes and in professing that their graduates are as competent as graduates from face-to-face programmes.

Standards for undergraduate e-learning programmes in the health professions should be distinct from face-to-face programmes. The adoption of standards of face-to-face programmes in undergraduate e-learning is detrimental to the development of e-learning in LMICs [26]. The need for standards for evaluating undergraduate e-learning programmes in the health professions is vital in ensuring quality health professionals. Further research should focus on quality standards in teaching and learning clinical skills in e-learning programmes.

## Conclusion

Learners graduating from e-learning and online programmes are currently viewed with scepticism, especially in terms of their clinical competence. However, the integration of digital technology in undergraduate education in the health professions has become inevitable. In most low-resource settings, face-to-face programmes remain superior and dominant as regulators and programme directors struggle to define and apply standards that comprehensively assess e-learning programmes’ quality. Delva et al. [1] noted that undergraduate e-learning programmes in the health professions must be comparable to face-to-face programmes. Still, caution should be taken regarding adopting face-to-face standards for online settings.

In this review, we synthesised standards that have been used for evaluating e-learning programmes in the health professions in LMICs to improve the quality of e-learning programmes. Only eight articles met the inclusion criteria–predominantly from the Middle East, North Africa and South-East Asia. Most of the standards described by the included articles aligned with popular models for evaluating e-learning programmes, with a few exceptions. A gap in clinical teaching and learning standards in undergraduate e-learning programmes in the health professions was evident in all the included articles.

Further research in this field should focus specifically on developing, expanding, and testing standards for evaluating the quality of e-learning that integrate teaching and learning of clinical skills. Such standards should allow evaluators to access clinical teaching sites, the educators within clinical teaching sites, and the nature and quality of clinical practice.

The contribution of this review is to identify themes used in evaluating the quality of undergraduate e-learning programmes in the health professions. The themes are curriculum planning, proficiency of the educator, learner proficiency and attitude, infrastructure for learning, support, and evaluation. When developing contextually appropriate interventions, it would be valuable to include teaching and learning of clinical skills.

## Limitations

Limitations of the review are, the search string and inclusion criteria, which may have excluded some studies. Literature sourced from was limited to English-language publications within LMICs. Abstracts and full texts in foreign languages were excluded. Articles only from the University of the Free state were accessed and this might have limited the scope. The risk of bias in each of the articles was not assessed. We acknowledge not conducting a methodological quality review might affect the study results and the potential of overestimating or underestimating findings, can inadvertently affect the quality of the study. However, rigorous screening and consensus discussion mitigated the issue.

## Supporting information

S1 Checklist PRISMA checklist. (DOCX)

S1 Text Phase 1 developed standards. (DOCX)

## List of abbreviations

LMICs: low- and middle-income countries
COVID 19: Coronavirus 2019
PRISMA: Preferred Reporting Items for Systematic Reviews and Meta-Analysis
CINAHL: Cumulative Index of Nursing and Allied Health Literature
