# Supplementary material for: Undergraduate e-learning programmes in health professions: An integrative review of evaluation standards in low- and middle-income countries
Source: PLoS One. 2023 Feb 13;18(2):e0281586. doi: 10.1371/journal.pone.0281586 (PMC9925085; doi:10.1371/journal.pone.0281586)
Supplement: S1 Text — (DOCX) [file pone.0281586.s002.docx]

STANDARDS FOR EVALUATING THE QUALITY OF UNDERGRADUATE NURSING E-LEARNING PROGRAMMES IN KENYA

M. Mutua &Dr. C.N. Nyoni

School of Nursing

University of the Free State

The Standard Domains

The six evaluation related domains identified were curriculum planning, educator proficiency, learner proficiency

Fig. 2 Standard broad domains

STANDARD 1: CURRICULUM PLANNING

Descriptor: Curricula is designed, implemented and evaluated to achieve proficiencies and objectives for the e-learning undergraduate nursing programme.

Criteria:

- 1. Wider consultation of stakeholders, including potential learners in the curriculum/programme development.
  2. Module content is aligned with national priorities and the clinical environment.
  3. Principles and guidelines for developing the course/curriculum are well known.
  4. Course material developed for the e-learning environment.
  5. Regular review of the programmes.
  6. A policy is available on teaching and learning strategies that employ a variety of
  7. innovative teaching approaches.
  8. The e-learning programme is approved by relevant internal and external structures that have approved programmes in the institution.
  9. Strategies are in place for regular monitoring of course plans and designs.
  10. Course plan and design reflect best e-learning practices.
  11. A comprehensive assessment policy should be integrated in the e-learning programme.
  12. Structures to enhance the security of online assessments.
  13. Assessment methods are aligned with best practices.
  14. Moderation of assessment practices.
  15. Learner satisfaction with the quality of assessments is considered.
  16. A system is available to enhance self-directness among all learners aligned with the

learning outcomes.

- 1. Learners have access to course content.
  2. Course content is current, regularly evaluated and updated.
  3. Educators are involved in course content development.
  4. There is evidence of developing problem-solving skills in learning and teaching material.
  5. Learners are facilitated on self-directed learning using electronic resources.
  6. Course material integrates authentic clinical examples.
  7. Various teaching modalities are integrated to support problem-solving.
  8. E-learning programme is socially responsive and accountable.
  9. E-learning programme policy, rules and regulations are available.
  10. E-learning programme policy, rules and regulations are explicit and available to educators and learners.
  11. E-learning programme goals are aligned with national and local statutory requirements.
  12. There is evidence of the integration of educational goals in the design and development of e-learning material.
  13. Educational goals are regularly reviewed and updated.
  14. E-learning programme provides opportunities for student collaboration

STANDARD 2: PROFICIENCY OF EDUCATOR

Descriptor: The quality of staff recruited in the programme, working conditions in place and the teaching strategy is adequately benchmarked, monitored and evaluated. Professional development approaches in place enable the programme to meet its stated objectives.

Criteria:

- 1. Educators on-job training on proficiency in the use of technology in education.
  2. Human resource (HR) policy supports the recruitment of educators with appropriate professional expertise for e-learning programmes.
  3. A strategy is available for Continuing Professional Development of technology enhanced education and facilitation of learning.
  4. Class size enables effective learning for each learner, utilising available teaching and learning resources for theoretical and clinical units.
  5. Institutional strategy for orientation and continuous support related to responsibilities, institutional expectations, and computer skills.
  6. Institutional strategy and policies should be in place to determine the e-learning needs of

educators.

- 1. Strategic plan for the institution/programme reflects the needs of educators related to facilitating e-learning.
  2. A strategy that monitors professionalism during class facilitation is available.
  3. All educators must be registered as professionals by a local professional body.
  4. Institutional policy to provide educators with access to various appropriate teaching resources, including innovative digital tools.
  5. Integrate learners’ knowledge of different digital tools in learning.
  6. Institutional policies that support equitable workload distribution allocation/ workload allocation model.
  7. Institutional strategy to provide minimum equipment required by educators.
  8. A strategy is in place for the evaluation of educator competence in facilitation.
  9. Institutional policy on e-learning-specific teaching methods, strategies, and approaches.
  10. Availability of teaching and learning resources, including timely access to information

technology (IT) facilities for educators and learners.

- 1. A system for monitoring and evaluating the teaching process and a feedback mechanism

to improve educator development initiatives.

- 1. The variety of teaching strategies are consistent with the profession's current

knowledge and fundamental theoretical aspects.

- 1. Learner evaluation of educator professionalism.

STANDARD 3: LEARNER PROFICIENCY AND ATTITUDE

Descriptor: Learners are provided with a variety of learning opportunities, empowered to develop resilience, reflective and caring attitudes to enable them achieve proficiencies in the e-learning programme and demonstrate professional behaviour.

Criteria:

- 1. A policy is available for learners to receive individual academic support based on needs, strength, skills, and interests.
  2. Learners are aware of the obligations and responsibilities of the programme at

registration.

- 1. Learner orientation on programme and technology requirements on admission.
  2. Learners are provided with course navigation options, an explanation of the purpose of

course material, how to navigate through the course, how to use the material and

expectations at the end of learning.

- 1. Library access to broader electronic resources and literacy skills required to handle these

resources appropriately.

- 1. Systems are available for learners to meet academic obligations before assessment.
  2. Learners enrolled in the e-learning programme have access to course materials,

educators and relevant technology, enabling them to succeed in the programme.

- 1. Technology-enhanced approaches integrate various apporaches to cater for the different

learning styles.

- 1. Opportunity for learners to measure their progress and satisfaction with the programme.
  2. Learning activities focus on the learners’ needs towards achieving measurable learning

outcomes.

- 1. Assessments are aligned with the learning outcomes.
  2. Technology, media and course tools support learning outcomes while incorporating

learner interests and guiding learners to active learning.

- 1. There is a system in place for the learners’ evaluation of the use of digital tools.
  2. Learner surveys during and at the course completion stage depict a robust e-learning

programme supporting learners’ interests..

STANDARD 4: INFRASTRUCTURE FOR LEARNING

Descriptors: Provision of human, financial technological, infrastructure and physical resources used in the delivery of the programme are suitable for the intended purpose and have the potential to enable the programme to achieve its stated objective.

Criteria:

4.1 Policies, plans, and structures ensure that the management and maintenance of

equipment and resources create an enabling environment for facilitating learning.

4.2 Availability of equipment to enable innovative teaching and learning methods.

4.3 Systems and sustainable funding are available for the regular maintenance and upgrading of infrastructure.

4.4 Accessible internet connectivity on and off campus.

4.5 A learning management system (LMS) is accessible on and off-campus.

4.6 Periodic updates to upgrade the quality of the LMS to ensure continued teaching and learning.

4.7 The curriculum clearly states minimum technology requirements and required technical

infrastructure.

4.8 Monitoring learner participation in the course to determine whether participation is

impeded due to technology and offer solutions.

4.9 Technological support is efficiently managed by qualified staff to ensure the operation of

technical aspects.

4.10 The institution’s policy on IT is sufficient to ensure its ability to plan, administer and

evaluate the e-learning programme.

4.11 Partnerships with relevant organisations to strengthen network infrastructure within the

organisation.

4.12 Learners have access to libraries’ ICT facilities during the day and after-hours supported

by competent staff.

4.13 A transparent appraisal system to enhance the performance of administrative and

support staff.

4.14 Provision of adequate space and buildings to accommodate teaching staff, management

staff, support staff and process of teaching and learning.

4.15 Policy to ensure that learning material developed by educators is protected under

intellectual property and educators’ terms for authorship and rights are agreed upon and

fulfilled.

4.16 All resources used in the course are appropriately cited and referenced.

4.17 A staff development system is in place for administrative and support staff to align with

best practices in e-learning.

4.18 The Learning management system and supports a variety of content material.

STANDARD 5: SUPPORT

Descriptors: Technical support for all actors to enable a conducive learning environment and attainment of the stated objectives of the programme.

Criteria

5.1 Technical support is provided for learners.
5.2 The course clearly articulates how to access help features for the course.
5.3 There is a system in place for regular training and support for learners on the use of the
learning management system.
5.4 The learners have access to learning, counselling and career services.
5.5 Support offered online provides learners with information on who to contact and the best
time to contact.
5.6 Support for educators to facilitate the learner-learner interactions by various modalities.
5.7 Remedial systems are in place to support learners who are not progressing as expected .
5.8 Monitoring students on e-learning platforms to ensure continuity and challenges
identified are resolved.
5.9 A complaint handling procedure exists and is well known to the learners and staff.

5.10 Continous professional development for educators on navigation challenges whilst
accessing, interacting and preparing assessments on the LMS.
5.11 Institutional policy to offer support to administrative staff in infrastructure development
to support the e-learning programme.
5.12 A system is in place to support learning for students during work-integrated learning
5.13 Institutional partnership with technological companies to ensure administrative staff
have skill development relevant to support e-learning.

STANDARD 6: EVALUATION

Descriptors: Programme evaluation is a sound, well-articulated procedure to ensure quality assurance, program effectiveness and compliance with regulation authorities to enhance programme delivery.

Criteria

6.1 Institutional procedures to monitor the use of learning infrastructure for computer and

internet usage within the institution is for learning purposes.

6.2 The Evaluation criteria of the e-learning programme implementation and strategic plan

are implemented.

6.3 The quality of the internet should support efficient and effective learning within online

media.

6.4 Learners fulfil all academic and clinical obligations before final summative assessments.

6.5 Ensure that learners evaluate learning, assessment techniques and technology required

for success in the e-learning programme.

6.6 Systems are available for regular review of programmes in consultation with stakeholders

and professional associations.

6.7 Institution to continuously improve e-learning programmes in terms of sufficient

investment in the course and material development, staff development and infrastructure

development.

6.8 Evaluation Information from all stakeholders in the e-learning programme provides

information for strengthening e-learning.

6.9 Demonstrate various evaluation methods that integrate information communication

technology (ICT) to enable learners to meet the programme learning outcomes.

6.10 Feedback solicited from students and gathered information is analysed for use in

systematic educator, learner, and administrative support staff development initiatives.
